# Dynamically-Driven Inactivation of the Catalytic Machinery of the SARS 3C-Like Protease by the N214A Mutation on the Extra Domain

**DOI:** 10.1371/journal.pcbi.1001084

**Published:** 2011-02-24

**Authors:** Jiahai Shi, Nanyu Han, Liangzhong Lim, Shixiong Lua, J. Sivaraman, Lushan Wang, Yuguang Mu, Jianxing Song

**Affiliations:** 1 Department of Biological Sciences, Faculty of Science, National University of Singapore, Singapore; 2 School of Biological Sciences, Nanyang Technological University, Singapore; 3 State Key Laboratory of Microbial Technology, Shandong University, Jinan, China; 4 Department of Biochemistry, Yong Loo Lin School of Medicine and National University of Singapore, Singapore; National Cancer Institute, United States of America and Tel Aviv University, Israel

## Abstract

Despite utilizing the same chymotrypsin fold to host the catalytic machinery, coronavirus 3C-like proteases (3CLpro) noticeably differ from picornavirus 3C proteases in acquiring an extra helical domain in evolution. Previously, the extra domain was demonstrated to regulate the catalysis of the SARS-CoV 3CLpro by controlling its dimerization. Here, we studied N214A, another mutant with only a doubled dissociation constant but significantly abolished activity. Unexpectedly, N214A still adopts the dimeric structure almost identical to that of the wild-type (WT) enzyme. Thus, we conducted 30-ns molecular dynamics (MD) simulations for N214A, WT, and R298A which we previously characterized to be a monomer with the collapsed catalytic machinery. Remarkably, three proteases display distinctive dynamical behaviors. While in WT, the catalytic machinery stably retains in the activated state; in R298A it remains largely collapsed in the inactivated state, thus implying that two states are not only structurally very distinguishable but also dynamically well separated. Surprisingly, in N214A the catalytic dyad becomes dynamically unstable and many residues constituting the catalytic machinery jump to sample the conformations highly resembling those of R298A. Therefore, the N214A mutation appears to trigger the dramatic change of the enzyme dynamics in the context of the dimeric form which ultimately inactivates the catalytic machinery. The present MD simulations represent the longest reported so far for the SARS-CoV 3CLpro, unveiling that its catalysis is critically dependent on the dynamics, which can be amazingly modulated by the extra domain. Consequently, mediating the dynamics may offer a potential avenue to inhibit the SARS-CoV 3CLpro.

## Author Summary

Severe acute respiratory syndrome (SARS) is the first emerging infectious disease of the 21st century which has not only caused rapid infection and death, but also triggered a dramatic social crisis. Its 3C-like protease is crucial for reproducing virus and thus represents a top target for drug design. Interestingly, unlike 3C protease such as from picorovirus, the SARS protease evolutionarily acquired a C-terminal extra domain with previously-unknown function. Immediately after SARS outbreak, we revealed that the extra domain was able to regulate the catalysis by controlling the dimerization essential for activity. Here, we studied one mutant with only slightly-weakened dimerization but almost completely abolished activity. We determined its three-dimensional structure but very unexpectedly it is almost identical to that of the wild-type enzyme. Therefore, we initiated 30-ns molecular dynamic simulations for five forms of the enzyme and the results demonstrate that the dynamical changes in this mutant are responsible for its inactivation. Therefore, the extra domain can also control the catalysis by modulating the enzyme dynamics. This is not only of fundamental significance to understanding how enzymes evolve, but also implies a novel avenue for design of anti-SARS molecules.

## Introduction

Severe acute respiratory syndrome (SARS) is the first emerging infectious disease of the 21st century and was caused by a novel coronavirus termed SARS-CoV. It suddenly broke out in China in 2002 and then rapidly spread to 32 countries, causing ∼8500 infections and over 900 deaths (http://www.who.int/csr/sars/en/). So far neither a vaccine nor an efficacious therapy has been available. Therefore, it remains highly demanded to design the potential therapeutic agents against SARS.

Coronaviruses are enveloped, positive-stranded RNA viruses with the largest single-stranded RNA genome (27–31 kilobases) among known RNA viruses. Its large replicase gene encodes two viral polyproteins, namely pp1a (486 kDa) and pp1ab (790 kDa), which are processed into active subunits for genome replication and transcription by two viral proteases [1], [2], namely the papain-like cysteine protease (PL2pro) and 3C-Like protease (3CLpro). Previously, SARS 3CLpro has been extensively considered to be a key target for development of antiviral therapies. The coronavirus 3CLpro, also known as main protease (Mpro), is so named to reflect the similarity of its catalytic machinery to that of the picornavirus 3C proteases [1]–[3]. Perceptibly, both 3C and 3CL-Like proteases utilize the two-domain chymotrypsin fold to harbor the complete catalytic machinery, which is located in the cleft between domains I and II [1], [3]. However, in the coronavirus 3CLpro, a ∼100-residue helical domain was additionally acquired at its C-terminus during evolution [1], [2], [4]. Moreover, unlike 3C protease, only the homodimeric form is catalytically competent for the SARS-CoV 3CLpro [1], [4], [5]–[20]. Immediately after the SARS outbreak, by a domain dissection approach, we have experimentally identified that the extra domain plays a key role in mediating the dimerization and intriguingly even the isolated extra domain could dimerize itself in solution [15]. Furthermore, detailed mutagenesis studies led to identification of different groups of extra-domain residues critical for both dimerization and catalysis [8], [12], [14], [20]. Now it is clear that both chymotrypsin fold and extra domain are critical for dimerization. Recently, we have succeeded in determining the high-resolution structure of R298A, a monomeric mutant in both solution and crystal [21]. In the R298A structure, the most affected regions are within the catalytic machinery, in addition to the several N- and C-tail residues as well as the orientation between the chymotrypsin fold and extra domain. Most importantly, we revealed that R298A has the completely collapsed and inactivated catalytic machinery structurally distinguishable from that in wild-type (WT) enzyme, characteristic of a chameleon formation of a short 3_10_-helix by residues Ser139-Phe140-Leu141 within the oxyanion-binding loop [21]. The collapsed catalytic machinery observed in R298A appears to represent a universal inactivated state intrinsic to all inactive enzymes because almost identical collapsed machinery was found in other two monomers, G11A and S139A, with the mutations located on the chymotrypsin fold [22], [23].

On the other hand, previously we also identified another mutant N214A, which owns a dramatically abolished activity but appeared to be largely dimeric as assessed by NMR spectroscopy. However, previous N214A construct contains two extra N-terminal residues Gly-Ser leftover from the thrombin cleavage of the GST-fusion protein. Since N-terminus has been shown to be critical for both activity and dimerization [17], in the present study, we generated the N214A enzyme without the two extra residues by reengineering the expression vector. Interestingly, the new N214A form only has a doubled dissociation constant (Kd) for the dimer-monomer equilibrium but almost abolished activity. Although initially we characterized it by NMR spectroscopy, many NMR peaks were undetectable because of its large size and conformational exchange on µs-ms time scale, thus preventing further characterization by NMR. As a result we subsequently determined the crystal structure of N214A but unexpectedly it adopts a dimeric structure almost identical to that of the WT protease. To explore whether the N214A mutation will trigger dynamical changes which account for the activity loss, we initiated 30-ns molecular dynamics (MD) simulations for the WT, N214A and R298A enzymes, as well as two artificial monomers derived from the dimeric WT and N214A structures. The obtained results unveil that for the SARS 3CLpro, the activated and inactivated states of the catalytic machinery are dynamically well separated. Very surprisingly, the N214A mutation triggers the dynamical instability of the catalytic machinery in the context of the dimeric form, with many key residues jumped to sample the conformations characteristic of the inactivated state.

## Materials and Methods

### Generation of the recombinant N214A mutant without any extra residue

Recently the extra N-terminal residues leftover from the cleavage of fusion proteins were demonstrated to significantly disrupt dimerization as well as to affect the enzymatic activity [17]. On the other hand, the SARS 3CLp we previously studied had two extra residues Gly-Ser after the thrombin cleavage of the GST-3CLp fusion proteins [14], [15]. In order to remove the two extra residues, in the present study we transferred the gene encoding SARS 3CLp from the pGEX-4T-1 GST-fusion expression vector (Amersham Biosciences, GE Healthcare, Little Chalfont, UK) to the His-tagged pET28a vector. Subsequently, site-directed mutagenesis was utilized to shorten the thrombin cleavage sequence LVPR|GS (CTG GTT CCG CGT GGA TCC) engineered by the company to LVPR| (CTG GTT CCG CGT), which only constituted the thrombin cleavage site in conjunction with the first two N-terminal residues Ser-Gly of SARS 3CLp. Interestingly, thrombin cleaved this new site (LVPR|SG) very efficiently to release the authentic wild-type 3CLpro. To produce N214A mutant, site-directed mutagenesis was further used to mutate Asn214 to Ala [14], [21]. Benefited from this re-engineered cleavage site, we were able to produce both authentic WT 3CLpro and its N214A mutant without any extra residues from the fusion tag. Recently, two structures were determined for the authentic SARS-CoV 3CLpro with PDB codes of 2H2Z [17] and 2GT7 [24].

The recombinant His-tagged WT and N214A proteases were expressed in *E. coli* strain BL21 (DE3) with induction by 0.4 mM isopropyl-1-thio-d-galactopyranoside (IPTG) at 20°C overnight. The WT and N214A proteases were obtained by affinity binding of the His-tagged proteins to the Nickel-NTA beads (QIAGEN), followed by the in-gel cleavage with thrombin to release the WT and N214A proteases, which was further purified by FPLC on a gel filtration column (HiLoad 16/60 Superdex 200). The molecular weight of the WT and N214A proteases were determined by a Voyager STR MALDI-TOF mass spectrometer (Applied Biosystems).

### Enzymatic activity assay and ITC characterization of dimerization

The enzymatic activities of the WT and N214A proteases were measured by a fluorescence resonance energy transfer (FRET)-based assay using a fluorogenic substrate peptide as previously described [13], [17], [25]. Briefly, the reaction mixture contained 50 nM protease and fluorogenic substrate with concentrations ranging from 1 µM to 30 µM in a 5 mM Tris-HCl buffer with 5 mM DTT at pH 6.0, which is identical to the crystallization condition. The enzyme activity was measured by monitoring the increase of the emission fluorescence at a wavelength of 538 nm with excitation at 355 nm using a Perkin-Elmer LS-50B luminescence spectrometer. The Km and kcat values were deduced from data analysis using Graphpad prism.

ITC experiments were carried out to determine the monomer-dimer dissociation constants of the WT and N214A proteases as previously described [7] using a Microcal VP ITC machine. Briefly, the protease samples and buffers were span at 13.3 k rpm for one hour to remove the tiny particles and degas thoroughly. In titrations, the WT or N214A sample in 5 mM Tris-HCl buffer at pH 6.0 containing 5 mM DTT were loaded in the syringe, which was subsequently titrated into the same buffer in the cell. The obtained titration data with endothermic peaks were analyzed by the built-in Microcal ORIGIN software using a dimer-monomer dissociation model to generate the dissociation constants and the enthalpy changes.

### Crystallization, structure determination of the N214A protease

The N214A protease with a concentration of 10 mg/ml was crystallized in a 2 µl hanging drop using a condition identical to that previously reported except for a minor variation of the PEG6000 concentration [4], [17]. The crystals were grown up to three days. 20% glycerol was supplemented with the mother liquid as a cryoprotectant. A single crystal was picked up from the cluster of crystals using the nylon loop and the X-ray diffraction data were collected at Bruker X8 PROTEUM in-house X-ray system.

The collected data set was processed using the program HKL2000 up to 2.3 Å resolution. The N214A was crystallized in the space group P21. The phase determination for the mutant structure was done by the molecular replacement method by using the WT SARS-CoV 3CLpro structure (PDB code: 2H2Z) as the searching model by the program Phaser [26] in the program suite Phenix [27]. The Ala mutating residues (Asn214) were corrected in the program COOT [28]. The refinements and the addition of the solvent molecules of the models for the mutant were done in the program suite Phenix [27]. The final model was analyzed by PROCHECK [29]. The data collection and refinement statistics are provided in the Table S1. The atomic coordinates have been deposited in the Protein Data Bank with the PDB code of 3M3S [30].

The structure overlay was done by LSQKAB from CCP4 suite [28]. All the figures were prepared using Pymol [31].

### Molecular dynamics (MD) simulations

The crystal structures of the WT (PDB code: 2H2Z) [17], the monomeric mutant R298A (PDB code: 2QCY) [21], and N214A determined in the present study were selected as the initial models for molecular dynamics simulations. In simulations, the WT and N214A proteases were in the dimeric forms while the R298A protease was a monomer. Furthermore, artificial monomers of WT and N214A obtained by splitting their dimeric structures were also subjected to the MD simulations under the same conditions.

The simulation cell is a periodic cubic box with a minimum distance of 10 Å between the protein and the box walls to ensure the protein would not directly interact with its own periodic image given the cutoff. The water molecules, described using the TIP3P model, were filled in the periodic cubic box for the all atom simulation. To neutralize the system, some Na+ and Cl- ions were randomly placed far away from the surface of the proteases. At the end, each system contained about more than 75,000 atoms.

Three independent 30-ns MD simulations were performed for each of five forms of the protease by the program GROMACS [32] with the AMBER-03 [33] all-atom force field. The long-range electrostatic interactions were treated using the fast particle-mesh Ewald summation method [34], with the real space cutoff of 9 Å and a cutoff of 14 Å was used for the calculation of van der Waals interactions. The temperature during simulation was kept constant at 300 K by Berendsen's coupling. The pressure was held at 1 bar. The isothermal compressibility was 4.5*10−5 bar-1. The time step was set as 2 fs. All bond lengths including hydrogen atoms were constrained by the LINCS algorithm [35]. Prior to MD simulations, all the initial structures were relaxed by 500 steps of energy minimization using steepest descent algorithm, followed by 100 ps equilibration with a harmonic restraint potential applied to all the heavy atoms of the protease.

## Results

### Activity and dimerization of the WT and N214A enzymes

By reengineering the thrombin cleavage site of the His-tagged pET28a vector, we succeeded in obtaining both WT and N214A proteases without any extra residues leftover from the fusion tag. The two enzymes were characterized by far-UV CD spectroscopy and their spectra (spectra not shown) had no detectable difference from those with two extra residues Gly-Ser that we previously studied, thus indicating that the two extra residues have no detectable effect on their secondary structures.

**Figure 1:**
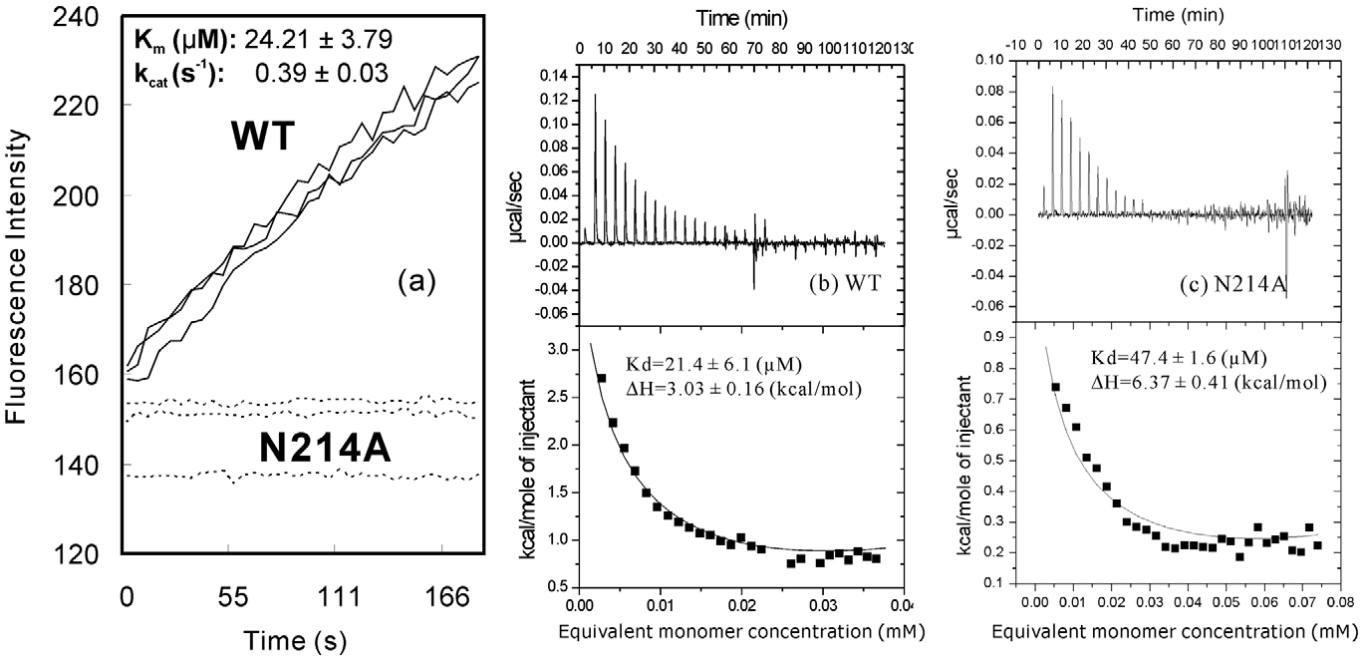
Enzymatic activity and dissociation constant of the dimer-monomer equilibrium. (a). Enzymatic activities of the WT (black lines) and N214A (dotted lines) proteases by monitoring the increase of the emission fluorescence intensity at a wavelength of 538 nm continuously for 3 min. The Km and kcat values are presented for the WT enzyme. The ITC dilution profiles for measuring the dissociation constants of the dimer-monomer equilibrium for WT (b) and N214A (c). The Kd and ΔH values were obtained by fitting the ITC data with the built-in Microcal ORIGIN software.

By a fluorescence resonance energy transfer (FRET)-based assay, we have measured the enzymatic activities of both WT and N214A proteases. As shown in Figure 1a, the WT protease is fully active with the Km and kcat values very similar to that previously reported on the authentic enzyme [17]. However, the activity of the N214A mutant is extremely low and consequently had no detectable increase of the fluorescence intensity within 3-minute incubation. Only after 2 hours, a slight increase of the fluorescence intensity could be detected (data not shown). We have also tested on the activity at higher N214A concentrations, no significant activity enhancement was observed. Because of this, we were unable to fit out their precise Km and kcat values although we collected a large set of data for N214A.

The dimerization of the SARS 3CLpro has been extensively characterized to be absolutely required for the enzymatic activity. As such, previously the dimer-monomer equilibrium has been exhaustively assessed by a variety of methods, including enzyme kinetics, chemical cross-linking, dynamic light scattering (DLS), isothermal titration calorimetry (ITC), analytic ultra-centrifuge (AUC) and small-angle X-ray scattering [5], [7]–[12], [14]. Interestingly, the obtained dissociation constant (Kd) values have an extremely-large variation, ranging from <1 nM to more than 200 µM in the literature. Recently, a study specifically aimed to reexamine these discrepancies by measuring the Kd values with three independent methods. Strikingly, the obtained Kd values for the WT SARS 3CLpro were very similar, 5.8–6.8 µM by small-angle X-ray scattering; 12.7 µM from chemical cross-linking and 5.2 µM from enzyme kinetics [10].

Previously, despite being catalytically active, the Kd value of a WT form of the SARS 3CLpro was derived to be 227 µM by using ITC method [7]. However, this protease form contained a 14-residue N-terminal tag with a sequence of MRGSHHHHHHGSTM. Here we used the same ITC method to measure the Kd values and the obtained values were 21.4 µM (Figure 1b) for the authentic WT protease and 47.4 for its N214A mutant (Figure 1c) respectively. Notably, the Kd value obtained here for the WT protease is ∼10-fold less than the previous one [7]. This difference may be attributed to the presence of the 14-residue His-tag in the protease previously characterized, as it is now well established that the presence of the extra N-terminal residues would significantly destabilizes the dimerization [10], [11], [17], [36]. Noticeably, the Kd value of the N214A mutant is only ∼2 fold higher, indicating that without the extra N-terminal tag residues, the N214A mutation itself has a very limited effect on the dimerization. On the other hand, the enzymatic activity of N214A is severely abolished. As a consequence, the observed loss of the N214A activity can not be completely attributed to the slight disruption of the dimerization. This conclusion is strongly supported by the fact that several forms of the enzyme containing different N-terminal tags have much more destabilized dimerization but still retain high enzymatic activity [4], [7], [9]–[11], [14], [17]. For example, the form with the extra 14 residues has a ∼10-fold increase of Kd value but still has very high enzymatic activity [7]. This result thus strongly implies that the extra domain may mediate the catalysis of the SARS 3CLpro by unknown mechanisms other than controlling the dimerization.

### Crystal structure of the N214A enzyme

To gain insights into the structural consequence of the N214A mutation, we determined its crystal structure at a resolution of 2.3 Å in P2_1_ space group. The R factor of the final model for N214A was 19.6%, with the Rfree factor of 25.7%. Details of the data collection and refinement statistics are presented in Table S1.

Remarkably, the N214A mutant still adopts the classic dimeric structure with the same packing of the two protomers as observed in all previously-determined dimeric structures of the coronavirus 3CLpro [1], [2], [4], [17], [18], [24], [31], [37]–[40]. In the electron density map of the N214A mutant, all residues are visible except for the last four residues Val303-Thr304-Phe305-Gln306 of the protomer B, which could not be identified due to the poorly defined electron densities.

**Figure 2:**
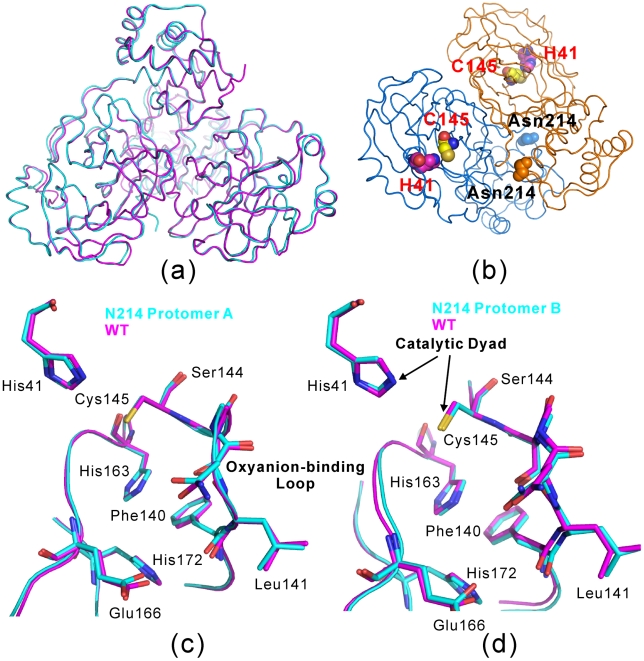
Crystal structure of the N214A mutant. (a). Overall superimposition of the dimeric N214A (violet) and WT (cyan; PDB code of 2H2Z) structures. (b) Dimeric structure of the SARS 3CLpro showing the catalytic dyad His41-Cys145 located on the cleft of domain I (red) and domain II (blue) of the chymotrypsin fold, as well as Asn214 on the extra domain. Superimpositions of the catalytically critical residues of WT (violet) and N214A (cyan; PDB code of 2H2Z) for protomers A (c) and B (d) respectively.

The dimeric N214A structure is highly similar to those of the WT protease previously reported (Figure 2). If compared to the WT crystal structure (2H2Z) with the authentic sequence in the C2 space group [17], the overall RMS deviation is 0.6 Å. A close examination reveals that the main difference is over the C-terminal 7 residues of the protomer A. Strikingly, if compared with another WT crystal structure (2GT7) also with the authentic sequence but in the P2_1_ space group [24], even the C-terminal residues have no marked difference and as a consequence the overall RMS deviation is only 0.2 Å. Interestingly, in the 2GT7 structure, the disordered regions include the last four residues Val303-Thr304-Phe305-Gln306 of the protomer B as well as additionally Thr45-Ala46-Glu47-Asp48-Met49 of the protomer A.

Unexpectedly, in the N214A mutant, even for the key residues constituting the catalytic machinery including the catalytic dyad His41-Cys145, oxyanion-loop Phe140-Cys145, His163 and Glu166 critical for binding substrates; and Phe140, His172 in holding the substrate binding-pocket open, their backbones and side-chains are almost superimposable to those in the WT structures (Figures 2c and 2d). This observation strongly implies that the loss of the N214A activity can not be readily rationalized only by the static structure. Instead, it may be due to the change of the dynamics of the enzyme as triggered by the N214A mutation. Therefore, we examined the B-factors of N214A, R298A and WT proteases (Figure S1) and indeed some regions in the N214A mutant do have higher B-factors. However, it appears not straightforward to establish a precise correlation between the B-factors and catalytic activity for the three enzymes.

### Molecular dynamics (MD) simulations

Molecular dynamics simulation is a powerful tool to pinpoint the dynamical factors underlying protein functions. To gain insights into their dynamical behaviors, we initiated 30-ns MD simulations for the WT, N214A and R298A, as well as artificial WT and N214A monomers. For R298A, we used the monomeric crystal structure we previously determined at 1.75 Å resolution [21]. For the WT enzyme, two structures with the authentic sequence are available: 2H2Z determined in C2 and 2GT7 in P2_1_ space group. Here, we selected the 2H2Z structure but not 2GT7 for MD simulations because residues Thr45-Met49 are missing in 2GT7.

**Figure 3:**
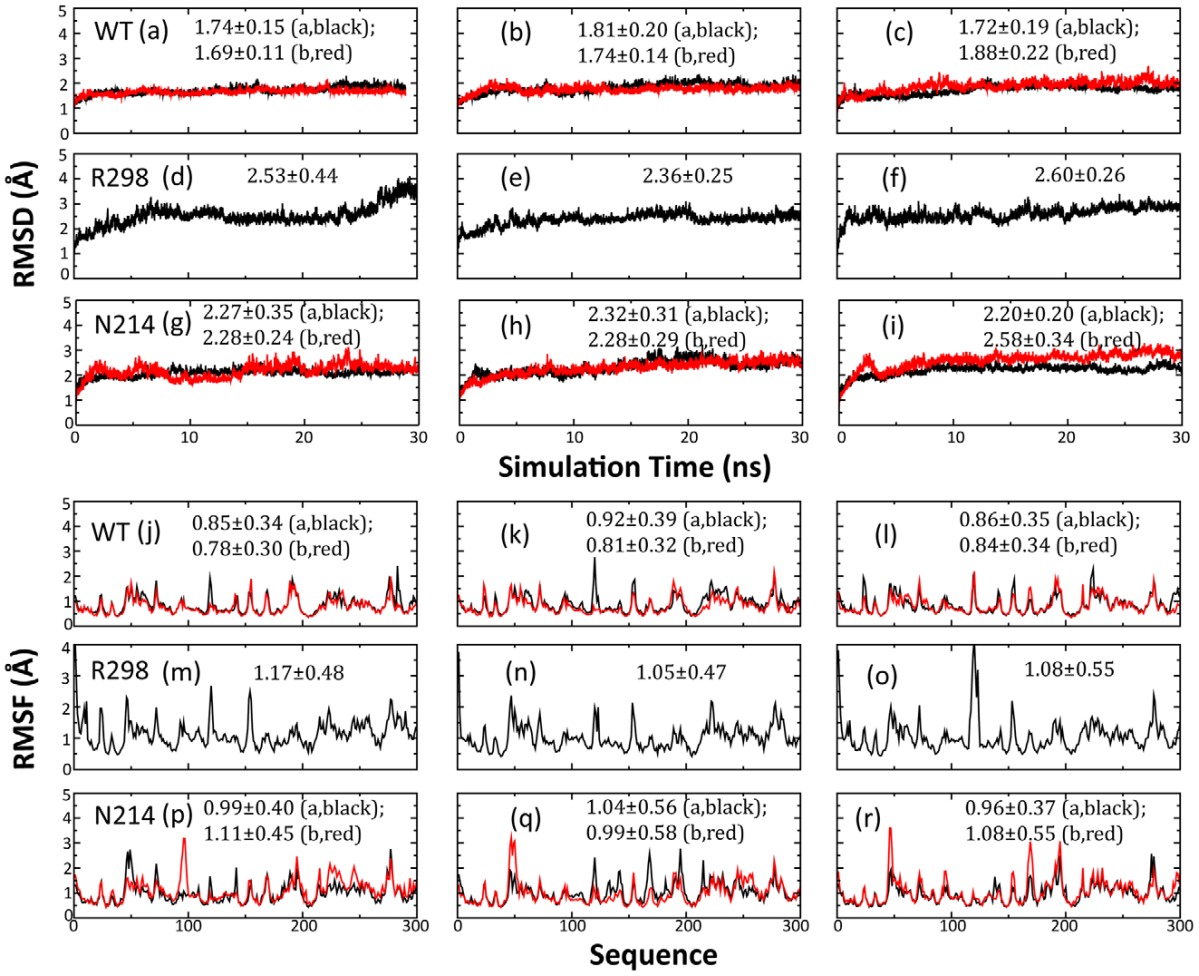
Overall dynamic behaviors in the MD simulations. Root-mean-square deviations (RMSD) of the heavy atoms for three independent MD simulations of the WT protomer A (black) and B (red) (a–c); R298A (black) (d–e); N214A protomer A (black) and B (red) (g–i). Root-mean-square fluctuations of the Cα atoms computed for three independent simulations for WT (j–l), R298A (m–o) and N214A (p–r).

Figure 3 presents the root-mean-square deviations (RMSD) of all heavy atoms for three parallel simulations for WT, R298A and N214A. It appears that for all simulations, the RMSD values increased very rapidly during the first 0.6 ns. This is mostly due to the relaxations of the crystal structures upon being solvated in solution as previously observed on MD simulations of the SARS 3CLpro [7], [22], [39], [41]–[43]. Very strikingly, three proteases display different dynamic behaviors. The WT enzyme appears to have the largest conformational rigidity, with the lowest RMSD values averaged over three simulations (1.75 and 1.76 Å for two protomers respectively). By contrast, R298A shows the highest overall conformational flexibility, with the largest average RMSD (2.50 Å). This feature may be largely due to the exposure of the interfacial residues to the solvent upon losing the counter monomer, as previously observed [22], [41], [43]. Indeed, much high RMSD values are observed for the simulations of the artificial WT and N214A monomers (Figure S2). More surprisingly, despite having the initial structure very similar to that of WT, both N214A protomers have much larger conformational flexibility than the WT enzyme, as clearly evidenced from their large average RMSD (2.20 and 2.34 Å for two protomers respectively). The RMSD values of the N214A dimer are also similarly larger than those of the WT dimer in three parallel simulations, implying no significant dissociation in N214A (Figure S3).

Noticeably, similar dynamic behaviors are also captured by the root-mean-square fluctuations (RMSF) of the Cα atoms in the MD simulations (Figures 3j–3r). R298A has the highest overall average RMSF value (1.10 Å) while both WT protomers have the lowest values (0.88 and 0.81 Å respectively). Although overall two N214A protomers have the average RMSF values (1.00 and 1.06 Å respectively) larger than two WT protomers but slightly smaller than R298A, it is noteworthy to point out that both N214A protomers have unusual large RMSF values over residues Ala46-Asn51. This region may be intrinsically flexible because residues Thr45-Met49 were totally disordered in the 2GT7 structure.

### Snapshots of the catalytic machinery in the simulations

We have extensively analyzed the conformational changes of individual residues in the MD simulations. It appears that the most dramatic and relevant changes are located within the catalytic machinery composed of the catalytic dyad and substrate binding subsite S1. As seen in Figure 2b, although the SARS 3CLpro acquires an extra domain at the C-terminus (domain III, residues 201–303), like the 3C protease it still uses the chymotrypsin fold made up of domains I (residues 8–101) and II (residues 102–184) to harbor the complete catalytic machinery, with the catalytic dyad His41-Cys145 and substrate binding-pocket located in a cleft between domains I and II. As a consequence, below we will be mostly focused on the analysis of the dynamical behaviors of the catalytic dyad and S1 substrate-binding subsite.

**Figure 4:**
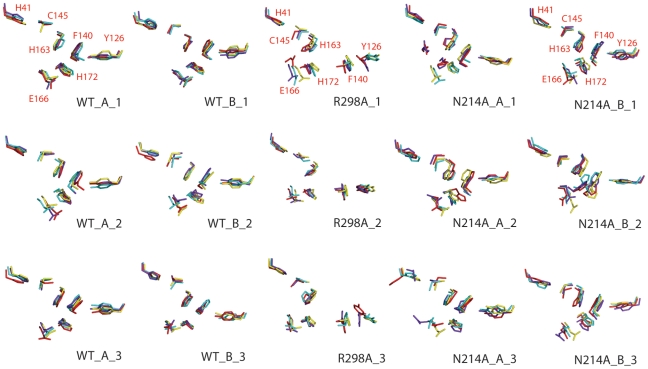
Structure snapshots in the MD simulations. The conformations of the residues constituting the catalytic machinery at 0 ns (yellow), 10 ns (cyan), 20 ns (blue) and 30 ns (red) for three independent simulations of WT, R298A and N214A respectively.

Figure 4 presents the structural snapshots for side chains of the key residues forming the catalytic dyad and S1 substrate-binding subsite in WT, N214A and R298A at the MD simulation time points of 0, 10, 20 and 30 ns. Overall in the both WT protomers, the conformational fluctuation of these side chains is relatively small except for that of Glu166. The relatively large fluctuation for Glu166 is understandable because it functions to bind the substrate but in the present simulations, the enzyme is not in complex with any substrate, unlike previous MD simulations [41], [43]. Consequently the Glu166 side chain is accessible to the bulk solvent and is expected to have a large fluctuation. By contrast, although N214A owns a crystal structure almost identical to that of WT, it has the much larger fluctuations in both protomers. On the other hand, in R298A due to the formation of a characteristic 3_10_-helix over residues Ser139-Phe140-Leu141, its catalytic machinery has become completely collapsed, as we previously demonstrated [21]. The collapsed machinery is structurally very different from that of the activated WT enzyme, as exemplified by the dramatic movements of the Phe140 and Tyr126 sidechains. Consequently the Phe140 aromatic ring only remains interacting with the Tyr126 aromatic ring but no longer interacts with the aromatic rings of His163 and His172. Amusingly, as shown in Figures 4, even with the simulation time up to 30 ns, the R298A catalytic machinery still remains highly trapped in the collapsed state.

### Dynamic behavior of the catalytic dyad

**Figure 5:**
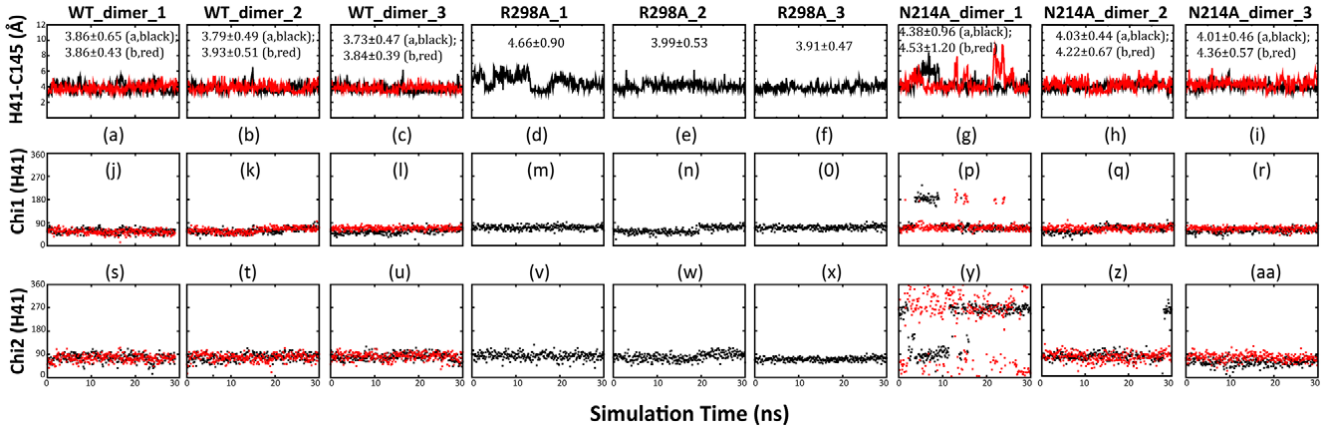
Dynamic behavior of the catalytic dyad. Time-trajectories of the distance between NE2 of His41 and SG of Cys145 atoms of WT (a–c); R298A (d–f) and N214A (g–i) in the 30-ns simulations. Time-trajectories of the Chi1 dihedral angle of His41 of WT (j–l); R298A (m–o) and N214A (p–r). Trajectories of the Chi2 dihedral angle of His41 of WT (s–u); R298A (v–x) and N214A (y–aa).

In the previously-determined crystal structures of SARS CoV 3CLpro, the distance between NE2 of His41 and SG of Cys145 ranges from 3.6 to 3.9 Å [43]. Furthermore, previous MD simulations also revealed that the dynamic stability of this distance is extremely critical for the stable formation of a hydrogen bond, which appears to be pivotal for maintaining the catalytic competency of the SARS 3CLpro [7], [22], [39], [41]–[43]. Also in previous MD simulations for the active WT enzyme, this distance has been demonstrated to range from 1.8–3.3 Å and 3.5–4.5 Å. Figure 5 presents the time-trajectories of this distance in the present simulations for WT, R298A and N214A. For the WT enzyme, the average value of the distance is 3.83 Å, while is 4.18 Å for R298A. Very surprisingly, for N214A, this distance has the largest average value of 4.26 Å. In particular, in the trajectory of one simulation (Figure 5g), there are several very large fluctuations. Consistent with this observation, the occupancy of the hydrogen bond between NE2 of His41 and SG of Cys145, which was calculated based on the dynamic behavior of both distance and angle, yields to 28.40% and 15.82% respectively for the WT protomers A and B; 5.42% for R298A, and 3.83%–8.55% respectively for the N214A protomers A and B. This result implies that only as judged from the occupancy of this hydrogen bond, the catalytic competency of both R298A and N214A enzymes is significantly inactivated.

We also analyzed the time-trajectories of the angles Chi1 (N-CA-CB-CG) and Chi2 (CA-CB-CG-CD2) of His41 for WT, R298A and N214A. Interestingly in both WT protomers, the Chi1 (Figures 5j–5l) and Chi2 (5s–5u) are dynamically stable. For R298A, the Chi1 (Figures 5m–5o) and Chi2 (5v–5x) also appears relatively stable. Very unexpectedly, in one simulation, both protomers of N214A, Chi1 (Figure 5p) and Chi2 (Figure 5y), are dynamically very unstable and jumping among several conformational clusters. Intriguingly, the instability observed in the simulations of the dimeric N214A largely disappears in those of the artificial N214A monomers (Figure S4).

### Dynamic behavior of the oxyanion hole

One key component of the catalytic machinery of the SARS 3CLp is the substrate-binding pocket composed of six subsites, namely S1–S6, corresponding to the P1–P6 residues of the substrate [1], [2], [4]. Out of them the S1 subsite is the most critical which confers an absolute specificity for a Gln at the P1 position of the substrate. As such, maintaining the intact conformation of S1 subsite is especially vital for catalysis. Briefly, the S1 substrate can be divided into four parts: the oxyanion hole, His163, Glu166 and Phe140 and its stabilizing elements [1], [2], [4], [23].

**Figure 6:**
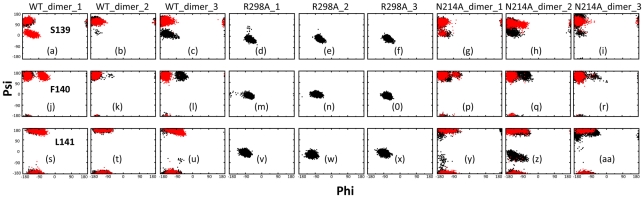
Dynamic behavior of the oxyanion loop residues. Ramachandran plots of the residues Ser139-Phe140-Leu141 for WT (a–c, j–l and s–u); R298A (d–f, m–o and v–x) and N214A (g–i, p–r and y–aa). The spots are colored in black for protomer A and red for protomer B.

The oxyanion hole refers to a structural element to donate two hydrogen bonds from main-chain amides of Gly-143 and Cys-145 to accommodate the main-chain oxygen of Gln-P1 as well as the tetrahedral intermediate during catalysis [1], [2], [4], [23]. Previously we demonstrated that in the inactive monomer R298A, the most distinguishable change is the collapse of the oxyanion hole as triggered by the chameleon formation of a short 3_10_-helix from a loop over residues Ser139-Phe140-Leu141 [21]. As seen in Figures 6a–6c, 6j–6l and 6s–6u, in WT these three residues mostly maintain the initial extended backbone conformations in the simulations. On the other hand, in R298A, even with the simulation time up to 30 ns, these residues also only sample the helical backbone conformations characteristic of the initial collapsed catalytic machinery in the R298A crystal structure. By contrast, in N214A, although residues Ser139-Phe140 sample the WT-like extended backbone conformations, Leu141 jumps to sample the helical conformation resembling that of R298A. Also it is worthy to point out that although the backbone conformations for the three residues of the artificial WT and N214A monomers are much more dynamic than those in the R298A monomer, they appears less dynamic than those for the dimeric N214A (Figure S5).

### Dynamic behavior of His163 and Glu166

The imidazole side chain of His163 plays a key role in determining the SARS 3CLpro specificity for glutamine at P1 by interacting with the P1 carboxamide side chain of the substrate. Previous MD simulations also showed that the dynamic behavior of the His163 side chain is critical for interacting with the substrate [7], [22], [39], [41]–[43]. Figure S6 presents the dynamic properties of the His163 backbones and side chains in WT, R298A and N214A. Interestingly, it appears that all three enzymes have the similar dynamic behaviors for the backbone conformations and Chi1 angles over the 30-ns simulations. Nevertheless, the Chi2 angles show some differentiations in three enzymes. In R298A, the His163 imidazole ring appears to flip rapidly among different conformations, probably due to the complete loss of the aromatic stacking interaction to the Phe140 ring in the collapsed catalytic machinery. On the other hand, in WT, the Chi2 angles only jumps to sample another conformational cluster. Interestingly, in all three N214A simulations, the Chi2 angle behaves much more dynamically than those in WT, as well as those in the artificial N214 monomer (data not shown).

**Figure 7:**
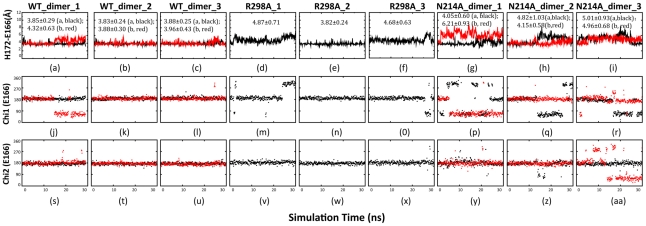
Dynamic behavior of the His172-Glu166 interaction. Time-trajectories of the distances between the aromatic rings of His172 and Glu166 of WT (a–c); R298A (d–f) and N214A (g–i) in three simulations. Time-trajectories of the Chi1 dihedral angle of Glu166 of WT (j–l); R298A (m–o) and N214A (p–r). Time-trajectories of the Chi2 dihedral angle of Glu166 of WT (s–u); R298A (v–x) and N214A (y–aa).

Glu166 is located at the entrance of the substrate binding pocket in the active enzyme, specifically recognizing the side-chain NE2 of Gln-P1. Figure 7 shows the dynamic behaviors of the distances between the Gln166 and aromatic ring of His172, as well as Chi1 and Chi2 angles of Gln166. Interestingly, WT has the shortest average distance (3.95 Å) and smallest fluctuations. By contrast, N214A has the highest fluctuations and consequently has the average distance (4.88 Å) even larger than that of R298A (4.46 Å). Also the Chi1 and Chi2 angles in three N214A simulations are the most dynamical. Interestingly again, the fluctuations reduce significantly for both the distance and angles in the simulations of the artificial N214A monomer (Figure S7).

### Dynamic behavior of Phe140 and other stabilizing factors

To maintain the catalytic machinery activated, Phe140 plays a key role by inserting its large aromatic ring into the S1 subsite to hold it open and active. The correct orientation of the Phe140 aromatic ring is maintained by the stacking interactions mainly with aromatic residues His163, His172 and Tyr126. Previously we have demonstrated that in the collapsed catalytic machinery of R298A, both backbone and aromatic ring of Phe140 underwent a remarkable movement and consequently Phe140 loses the stacking interactions with His163 and His172 [21].

**Figure 8:**
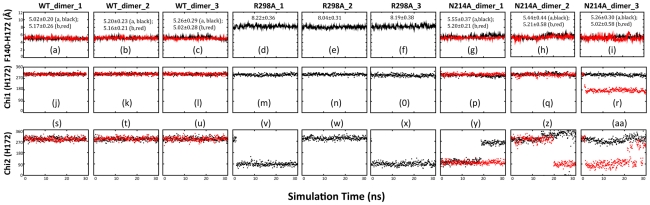
Dynamic behavior of the Phe140-His172 interaction. Time-trajectories of the centroid distances between the aromatic rings of Phe140 and His172 of WT (a–c); R298A (d–f) and N214A (g–i) in three independent simulations. Time-trajectories of the Chi1 dihedral angle of His172 of WT (j–l); R298A (m–o) and N214A (p–r). Time-trajectories of the Chi2 dihedral angle of His172 of WT (s–u); R298A (v–x) and N214A (y–aa).

Figure 8 shows the dynamical behaviors for the aromatic interaction between Phe140 and His172. In WT, the centroid distance remains short and dynamically stable in three simulations, with average values of 5.16 and 5.12 Å respectively for two protomers. By contrast, in R298A, this distance remains large as observed in the crystal structure, with an average value of 8.15 Å, indicating the total absence of this stacking interaction in the whole 30 ns simulations. For N214A, the dynamic behavior of this distance is very similar to that for WT, despite having slightly larger average values of 5.42 and 5.14 Å respectively for two protomers. As for the side chain conformations, the His172 Chi1 angles have both a very similar value as well as similar dynamical behavior in all three enzymes except that in one simulation of N214A, one protomer jumps to sample another conformation (Figure 8r). However, significant dynamics are observed for the N214A Chi2, which jumps to sample several conformational clusters in simulations (Figures 8y–8aa).

### Dynamic behavior of the mutation site and other relevant residues

In addition to the residues constituting the catalytic machinery, we have also extensively analyzed the dynamic behaviors of other residues in the MD simulations. The majority of them have similar values as well as dynamic behaviors in WT, R298A and N214A. For example, even for Tyr126 directly involved in interacting with Phe140, it has very similar conformations and dynamic behaviors for its backbones and side chains in WT, R298A and N214A (data not shown).

**Figure 9:**
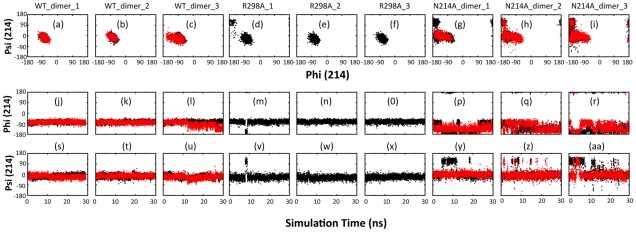
Dynamic behavior of the mutation site. Ramachandran plots of the residues Asn214 for WT (a–c); R298A (d–f) and Ala214 of N214A (g–i) in the 30 ns simulations. Time-trajectories of the Phi dihedral angle of Asn214 of WT (j–l); R298A (m–o), and Ala214 for N214A (p–r). Time-trajectories of the Psi dihedral angle of Asn214 of WT (s–u); R298A (v–x), and Ala214 for N214A (y–aa).

Nevertheless, although in the crystal structures, the backbones of the residues Asn214 (in WT) and Ala214 (in N214A) have very similar conformations, they display distinctive dynamic behaviors in the simulations (Figure 9). For both WT protomers as well as R298A, the Phi and Psi angles remain dynamically stable, only with slight fluctuations. By a sharp contrast, the backbone conformation of Ala214 in N214A becomes highly dynamic in all three simulations (Figures 9p–9r and 9y–9aa). However, the high dynamics of the Ala214 backbone disappear in the simulations of the artificial N214A monomer (Figure S8).

In the crystal structure of the WT enzyme, Asn214 is located at the end of the first helix of the extra domain which is sitting in between the N-finger and C-tail of the same protomer (Figure 2b). Its side-chain ND atom forms a hydrogen bond with the Gly2 backbone oxygen atom. Although no significant conformational change over these regions was detected in the crystal structure of the N214A mutant, the replacement of Asn214 by Ala did result in the elimination of the hydrogen bond between Asn214 and Gly2. Furthermore, we have calculated the hydrogen bond occupancy in the simulations and found that indeed in WT, the Asn214 was able to dynamically establish a variety of long-range intra-protomer hydrogen bonds with N-terminal residues Ser1 and Gly2, as well as with C-terminal residues Cys300 in both protomers, with some having relatively large average occupancies. By contrast, in N214A, only one long-range intra-protomer hydrogen bond could be found between the Gly2 backbone NH and Ala214 backbone oxygen atoms, with only an average occupancy of 2.85%. This suggests that in N214A, the packing would be weakened between the N- and C-termini, as well as between the N-finger with the rest part of the protein within the same protomer. On the other hand, previous studies have elegantly revealed the extremely critical involvement of the N-finger residues in both dimerization and catalysis due to their extensive contacts with residues composed of the active-site pocket of the counter protomer, which include Lys137-Phe140, Glu166, His172 [4], [7], [11], [12], [14], [17], [24].

Here we further analyzed the occupancy of the inter-protomer hydrogen bonds associated with the N-finger residues. Strikingly, in the simulations, the N-finger residues in N214A form more hydrogen bond contacts with the active-site residues in the counter protomers which also have higher occupancy than those in WT (Table S2). Also it appears that the backbone conformations of the N-finger residues in N214A are slightly more dynamic than in WT (Figure S9). These observations together may rationalize why the Asn214Ala mutation only slightly weakens the dimerization but drastically inactivates the catalysis. It might be likely that upon replacing Asn214 with Ala, the hydrogen bonds of Asn214 with the N- and C-termini in the same protomer are mostly eliminated. As a result, the N-finger residues would be liberated to some degree from interacting with the residues in the same protomer and as such have more capacity to contact the active-site residues in the counter protomer, as exemplified by the increased formation of hydrogen bonds between them. This enhanced inter-protomer interaction between the N-finger and active-site residues might cause some dynamical rearrangements of the active-site residues which may trigger the dynamical instability of the catalytic machinery, as captured by the above analysis.

On the other hand, the N214A mutation may also have some globular effect as to slightly weaken the dimerization and to provoke the dynamics of the residues Ala46-Asn51. These effects may also play additional roles in destabilizing the catalytic machinery. Our present results nicely agree with the previous observation that despite being highly conserved among different coronaviruses, the IBV 3C-like protease has Ser instead of Asn in the corresponding position of Asn214 but it still has a dimeric structure [44]. In the future, it would be interesting to investigate whether this substitution in the context of the IBV protease will weaken the intra-protomer interactions associated with the N-finger residues, and if yes, how the enzyme evolves the mechanism in IBV to prevent the active-site pocket being significantly destabilized.

## Discussion

By acquiring additional non-catalytic domains during evolution, enzymes have been shown to gain altered catalytic mechanisms or/and be connected to cellular signaling networks [45], [46]. Indeed, upon having the C-terminal extra domain, the SARS-CoV 3CLpro suddenly requests the dimerization to activate its catalysis. By contrast, the monomeric form is enzymatically inactive because its catalytic machinery is collapsed into the highly-conserved inactivated state in all monomeric structures [21]–[23]. Previously, our determination of the monomeric R298A structure reveals how the extra domain controls the dimerization which is ultimately coupled to the catalysis [21]. Recently, the isolated C-terminal domain has been found to form a domain-swapped dimer which may underlie the formation of the intermediate dimer structurally differential from the classic one [19], [47]. Notably, this non-classic dimer was recently proposed to be capable of performing N-terminal autocleavage which might mimic the initial autocleavage of the proprotein *in vivo* [48].

On the other hand, previously we have also identified N214A, another mutant which had significantly abolished activity but appeared to remain highly dimeric by our NMR characterization. In the present study, we examined the new version of the N214A mutant without the two extra residues leftover from the GST-fusion protein. Similarly, the new N214A protease again owns severely abolished activity but its dimer-monomer dissociation constant only slightly increased, from 21.4 to 47.4 µM. As such, it appears that in addition to modulating the dimerization, the extra domain may be able to regulate the catalysis by other unknown mechanisms. In an attempt to understand its structural basis, we determined the crystal structure of the N214A mutant and unexpectedly it still adopts a dimeric structure highly similar to that of the wild-type enzyme. In particular, N214A has the catalytic machinery almost identical to that of the wild-type enzyme. This result thus raises an intriguing question as how the N214A mutation on the extra domain is able to dramatically inactivate the catalytic machinery without significantly affecting its three dimensional structure.

The dynamics of the enzyme molecules have been extensively revealed to modulate the catalysis [49]–[53]. As such, we conducted 30-ns MD simulations for the WT, R298A and N214A enzymes, which, to the best of our knowledge, represent the longest MD simulations for the SARS-CoV 3CL proteases reported so far. Detailed analysis reveals that the three enzymes have very distinctive dynamic behaviors in the simulations. Although in the present study, the simulation time increased up to 30 ns, the WT enzyme not only displays the highest overall dynamic stability, but also has the catalytic machinery highly retained in the activated state, completely consistent with the previous MD results [7], [22], [39], [41]–[43]. On the other hand, despite showing an increased overall conformational flexibility of R298A, largely due to the additional exposure of interfacial residues to bulk solvent upon losing the counter protomer, as evidenced by the increase of the RMSD values in the simulations of the artificial WT and N214A monomers, the catalytic machinery of R298A remains largely trapped in the inactivated state. This result strongly implies that for the catalytic machinery of the SARS-CoV 3CLpro, the activated and inactivated states are not only structurally distinguishable, but also dynamically well separated. As a result, upon losing the dimerization, the catalytic machinery will be collapsed and subsequently permanently frozen in the inactivated state as we previously proposed [21]. Furthermore, our MD simulations also reveal the first dynamic picture of an experimentally-determined monomer of the SARS-CoV 3CLpro, which is slightly different from the previous [41], [43] as well as our present MD simulation results for two artificial monomers obtained by simply removing the counter protomer from the WT and N214A dimeric structures. In those simulations, the artificial WT and N214A monomers showed more dynamical instability than R298A for some key components of the catalytic machinery. The discrepancy may be mainly due to the possibility that in a 10-ns period, the artificial monomers with an initial WT structure could not even reach the real monomeric structure, which has marked changes in C-, N-termini and catalytic machinery, as well as the orientation between the extra domain and the chymotrypsin fold [21]–[23].

Most surprisingly, although the N214A mutant has the three-dimensional structure highly similar to that of the WT enzyme, in the simulations it has much larger conformational fluctuations than WT and its catalytic machinery is even more unstable than that of the R298A mutant and its own artificial monomer. In particular, during the simulations, the key distance between His41 and Cys145, which has been characterized to be an indicator of the competency of the catalytic dyad [7], [22], [39], [41]–[43], displays the largest fluctuation in the N214A mutant. This implies that even only based on the dynamic behavior of the catalytic dyad, the N214A catalytic machinery is largely inactivated. Furthermore, many N214A residues made up of the catalytic machinery are dynamically unstable and some even jump to sample the conformations resembling those of R298A. Therefore, even within the 30-ns simulation, the N214A mutant already displays dynamically unstable behaviors which may at least partly rationalize the observed abolishment of the catalytic activity. Furthermore, it is possible that more dynamical changes would be disclosed by MD simulations with longer simulation times such as over µs to ms.

Previously, it has been documented that mutations far away from the active site were able to affect the catalysis by triggering long-range dynamical changes but it remains extremely challenging to define the exact pathway of the transmission of dynamics [9], [50], [52], [53]. In the case of N214A, we show that the mutation of Asn214 to Ala will lead to eliminating most of the hydrogen bonds between the Asn214 and N-/C-terminal residues within the same protomer, as observed in the crystal structures as well as captured by the MD simulations. As a consequence, the N-finger residues would have more capacity to contact the key residues constituting the catalytic machinery of the counter protomer, such as Lys137, Gly138, Ser139, Phe140, Glu166 and His 172. It seems that the enhanced interaction in N214A may act as a perturbation which significantly triggers the dynamical instability of the catalytic machinery, which ultimately leads to the severe abolishment of the catalytic activity. In this regard, the N-finger appears to play dual roles in regulating the catalytic machinery. Namely, the maintenance of the competent catalytic machinery absolutely needs the supportive interactions from the N-finger residues of another protomer as previously well demonstrated [4], [7], [11], [12], [14], [17], [21]–[24]. Nevertheless, if these interactions are overwhelming, the catalytic machinery would also be dynamically destabilized. It is possible that the slight weakening of the dimerization in N214A may contribute to the observed instability of its catalytic machinery to some degree. However, this contribution may not be significant because within the present 30-ns simulations, no obvious dissociation of two protomers occurs, as judged from the comparison of the RMSD values for the WT and N214A dimers, as well as the fact that even more inter-protomer hydrogen bonds with high occupancy are found over the key dimerization interface, namely between the N-finger and active-site residues of N214A. In particular, the artificial N214A monomer with the dimerization interface fully exposed to solvent has much less fluctuations than the N214A dimer for the trajectories of most key residues constituting the catalytic machinery.

In conclusion, our present experimental and MD simulation studies unveil that the activated and inactivated states of the catalytic machinery of the SARS-CoV 3CLpro are not only structurally distinctive, but also dynamically separated. Furthermore, although the N214A mutation only slightly disrupts the dimerization and has no notable alteration on the static three-dimensional structure, it does trigger the dynamic instability of the catalytic machinery, thus rationalizing the severe abolishment of the enzymatic activity. Our current study thus signifies the potential to pinpoint the dynamical consequence of the mutations on the enzymatic catalysis by a combined use of experimental and computational approaches. Furthermore, the present results imply that to modulate the dynamics of the SARS-CoV 3CLpro may represent a promising avenue for design of its inhibitors for the anti-SARS therapeutics. In the future, it would be of fundamental interest to test this possibility by using NMR or/and Mass spectrometry to identify compounds which can significantly enhance the dynamics of enzymes and subsequently to correlate their effect on catalysis to the dynamic alteration.

## Supporting Information

Figure S1 Experimentally-derived B factors for the crystal structures of the N214A protomer A (a), protomer B (b); R298A (c) and WT (d, PDB code of 2H2Z).
(8.79 MB TIF)

Figure S2 Overall dynamic behaviors of three monomers. Root-mean-square deviations (RMSD) of the heavy atoms for three independent MD simulations of the artificial WT (a); R298A (b) and artificial N214A monomers (c). Root-mean-square fluctuations of the Ca atoms computed for three simulations of the artificial WT (d); R298A (e) and artificial N214A monomers (f).
(0.94 MB TIF)

Figure S3 Overall dynamic behaviors of WT and N214A dimers. Root-mean-square deviations (RMSD) of the heavy atoms for three independent simulations for the WT (a-c) and N214A (d-f) dimers.
(5.59 MB TIF)

Figure S4 Dynamic behavior of the catalytic dyad in three monomers. Time-trajectories of the distance between NE2 of His41 and SG of Cys145 atoms of the artificial WT (a–c); R298A (d–f) and artificial N214A (g–i) monomers in three independent simulations. Time-trajectories of the Chi1 dihedral angle of His41 of the artificial WT (j–l); R298A (m–o) and artificial N214A (p–r) monomers. Trajectories of the Chi2 dihedral angle of His41 of the artificial WT (s–u); R298A (v–x) and artificial N214A (y–aa) monomers.
(4.22 MB TIF)

Figure S5 Conformations of the oxyanion-loop in three monomers. Ramachandran plots of the oxyanion-loop residues Ser139-Phe140-Leu141 in three independent simulations for the artificial WT, R298A and artificial N214A monomers.
(4.37 MB TIF)

Figure S6 Dynamic behavior of His163. Ramachandran plots of His163 of WT (a–c); R298A (d–f) and N214A (g–i) in three independent simulations. Time-trajectories of the Chi1 dihedral angle of His163 of WT (j–l); R298A (m–o) and N214A (p–r). Trajectories of the Chi2 dihedral angle of His163 of WT (s–u); R298A (v–x) and N214A (y–aa).
(0.34 MB TIF)

Figure S7 Dynamic behavior of the His172-Glu166 interaction in three monomers. Time-trajectories of the distances between the aromatic rings of His172 and Glu166 of the artificial WT (a–c); R298A (d–f) and artificial N214A (g–i) monomers in three independent simulations. Time-trajectories of the Chi1 dihedral angle of Glu166 of the artificial WT (j–l); R298A (m–o) and artificial N214A (p–r) monomers. Time-trajectories of the Chi2 dihedral angle of Glu166 of the artificial WT (s–u); R298A (v–x) and artificial N214A (y–aa) monomers.
(4.11 MB TIF)

Figure S8 Dynamic behavior of the mutation site in three monomers. Ramachandran plots of the residues Asn214 for the artificial WT (a–c); R298A (d–f) and Ala214 for the artificial N214A (g–i) monomers in three independent simulations. Time-trajectories of the Phi dihedral angle of Asn214 of the artificial WT (j–l); R298A (m–o), and Ala214 for the artificial N214A (p–r) monomers. Time-trajectories of the Psi dihedral angle of Asn214 of the artificial WT (s–u); R298A (v–x), and Ala214 for the artificial N214A (y–aa) monomers.
(4.65 MB TIF)

Figure S9 Ramachandran plots of the N-finger residues Gly2-Met6 in the three independent simulations for WT, R298A and N214A.
(0.28 MB TIF)

Table S1 Data collection and refinement statistics of the N214A crystal structure.
(0.05 MB PDF)

Table S2 Average Occupancy (%) of hydrogen bonds associated with Asn214 and N-finger in WT/N214A.
(0.06 MB PDF)
