# Supplementary figures and images for: Dynamically-Driven Inactivation of the Catalytic Machinery of the SARS 3C-Like Protease by the N214A Mutation on the Extra Domain
Source: PLoS Comput Biol. 2011 Feb 24;7(2):e1001084. doi: 10.1371/journal.pcbi.1001084 (PMC3044768; doi:10.1371/journal.pcbi.1001084)

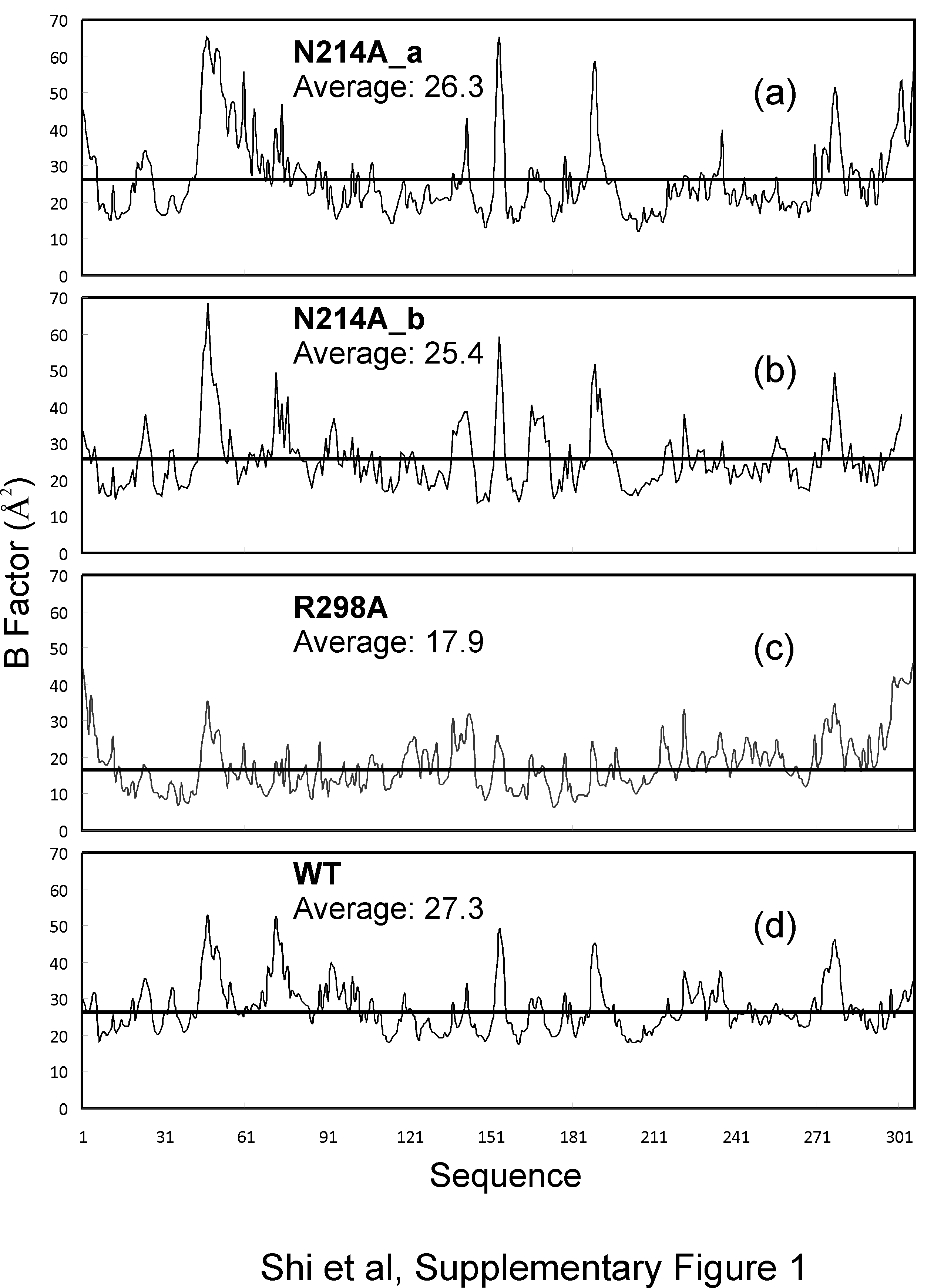

Supplement: Figure S1 — Experimentally-derived B factors for the crystal structures of the N214A protomer A (a), protomer B (b); R298A (c) and WT (d, PDB code of 2H2Z). (8.79 MB TIF) [file pcbi.1001084.s001.tif]

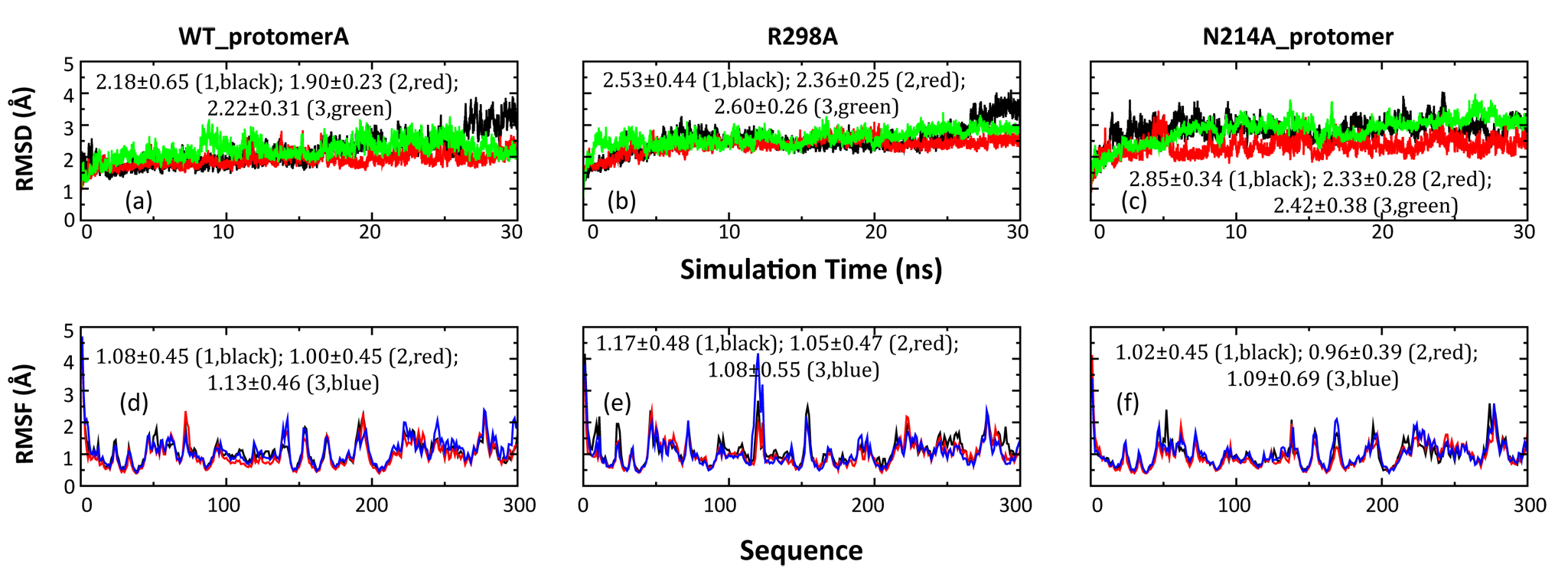

Supplement: Figure S2 — Overall dynamic behaviors of three monomers. Root-mean-square deviations (RMSD) of the heavy atoms for three independent MD simulations of the artificial WT (a); R298A (b) and artificial N214A monomers (c). Root-mean-square fluctuations of the Ca atoms computed for three simulations of the artificial WT (d); R298A (e) and artificial N214A monomers (f). (0.94 MB TIF) [file pcbi.1001084.s002.tif]

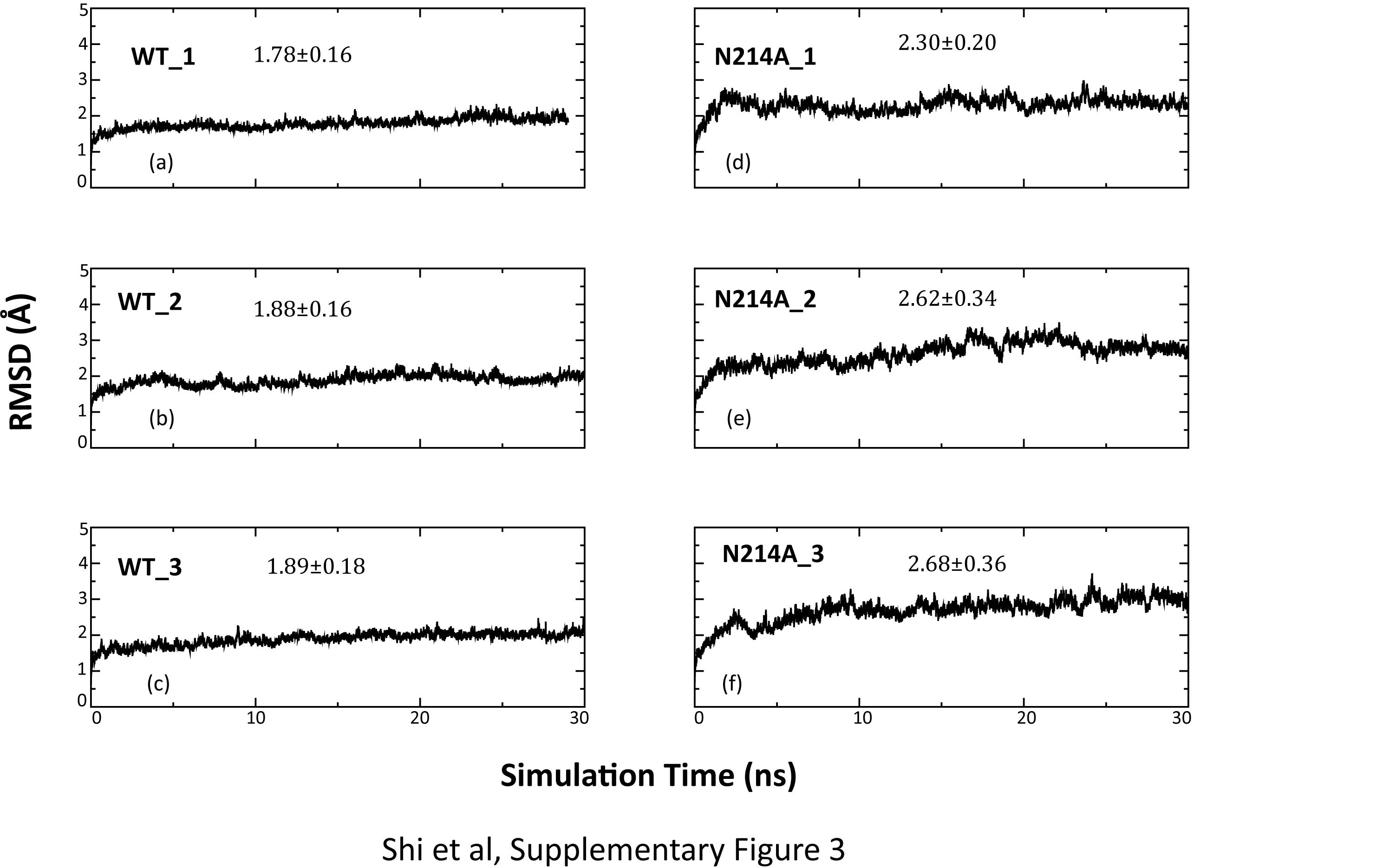

Supplement: Figure S3 — Overall dynamic behaviors of WT and N214A dimers. Root-mean-square deviations (RMSD) of the heavy atoms for three independent simulations for the WT (a-c) and N214A (d-f) dimers. (5.59 MB TIF) [file pcbi.1001084.s003.tif]

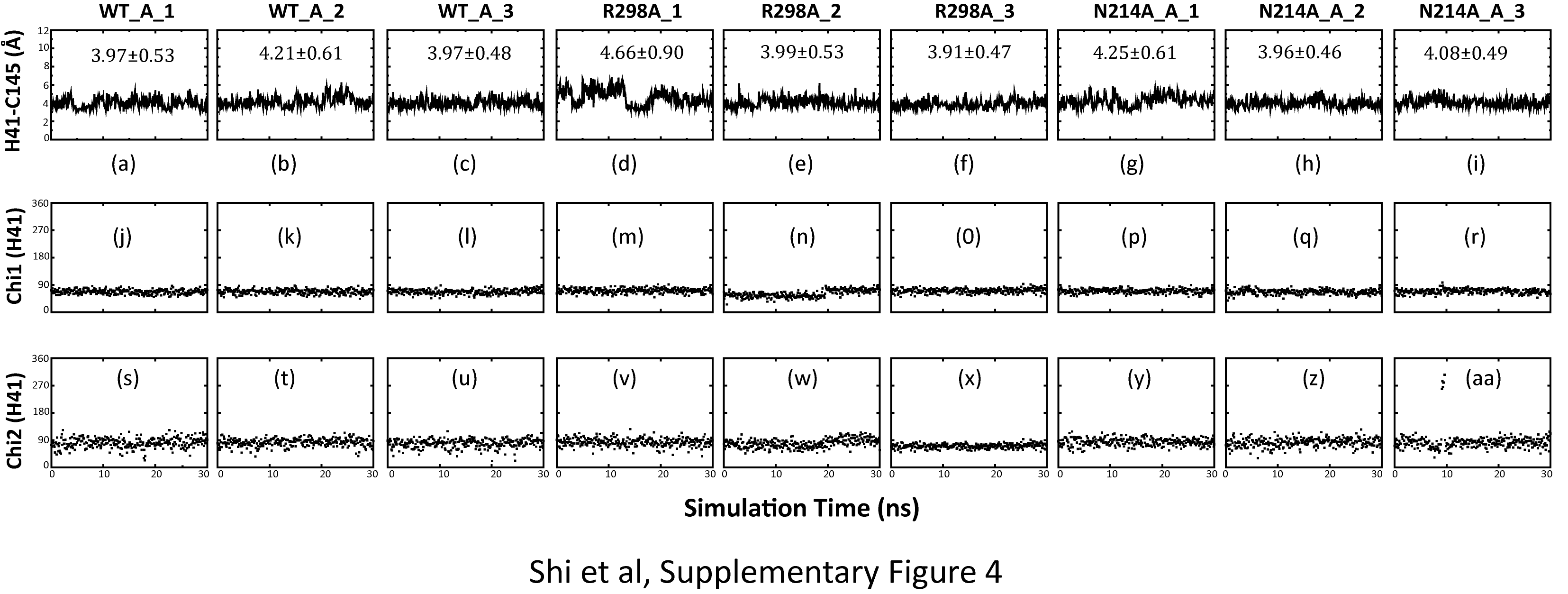

Supplement: Figure S4 — Dynamic behavior of the catalytic dyad in three monomers. Time-trajectories of the distance between NE2 of His41 and SG of Cys145 atoms of the artificial WT (a–c); R298A (d–f) and artificial N214A (g–i) monomers in three independent simulations. Time-trajectories of the Chi1 dihedral angle of His41 of the artificial WT (j–l); R298A (m–o) and artificial N214A (p–r) monomers. Trajectories of the Chi2 dihedral angle of His41 of the artificial WT (s–u); R298A (v–x) and artificial N214A (y–aa) monomers. (4.22 MB TIF) [file pcbi.1001084.s004.tif]

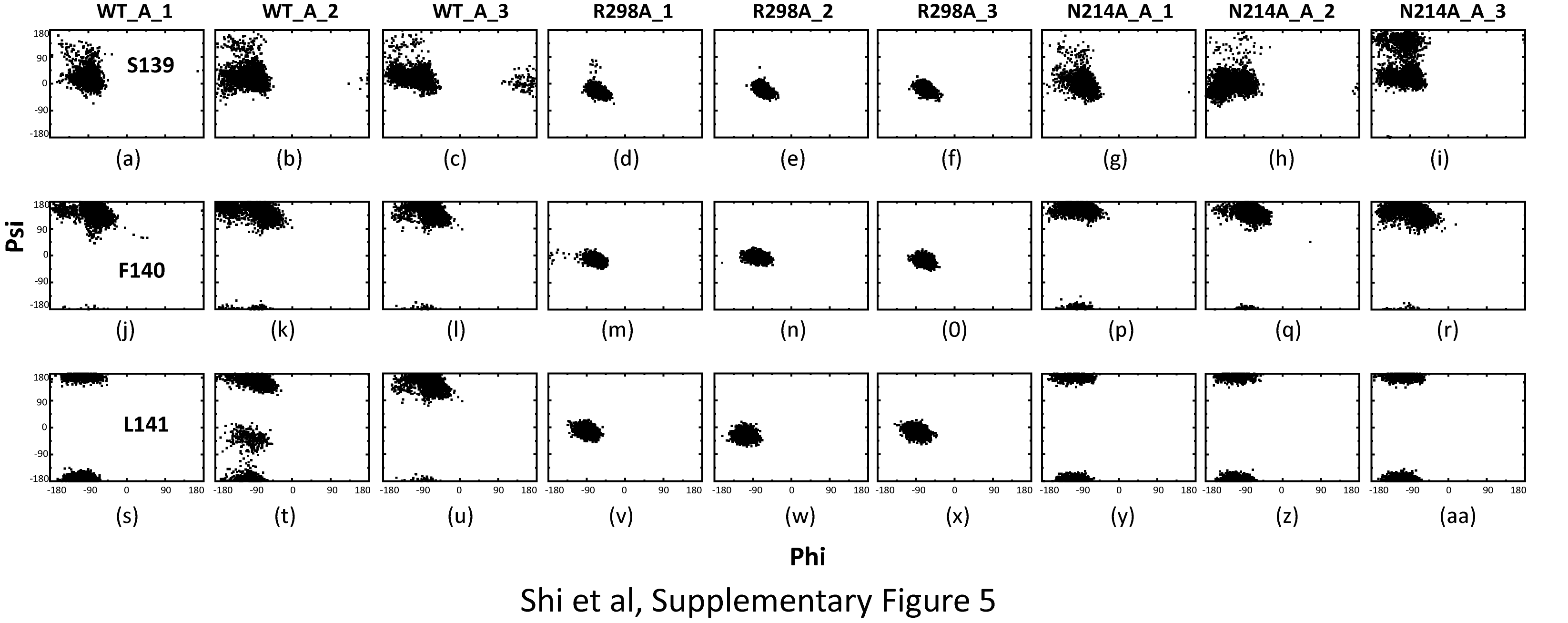

Supplement: Figure S5 — Conformations of the oxyanion-loop in three monomers. Ramachandran plots of the oxyanion-loop residues Ser139-Phe140-Leu141 in three independent simulations for the artificial WT, R298A and artificial N214A monomers. (4.37 MB TIF) [file pcbi.1001084.s005.tif]

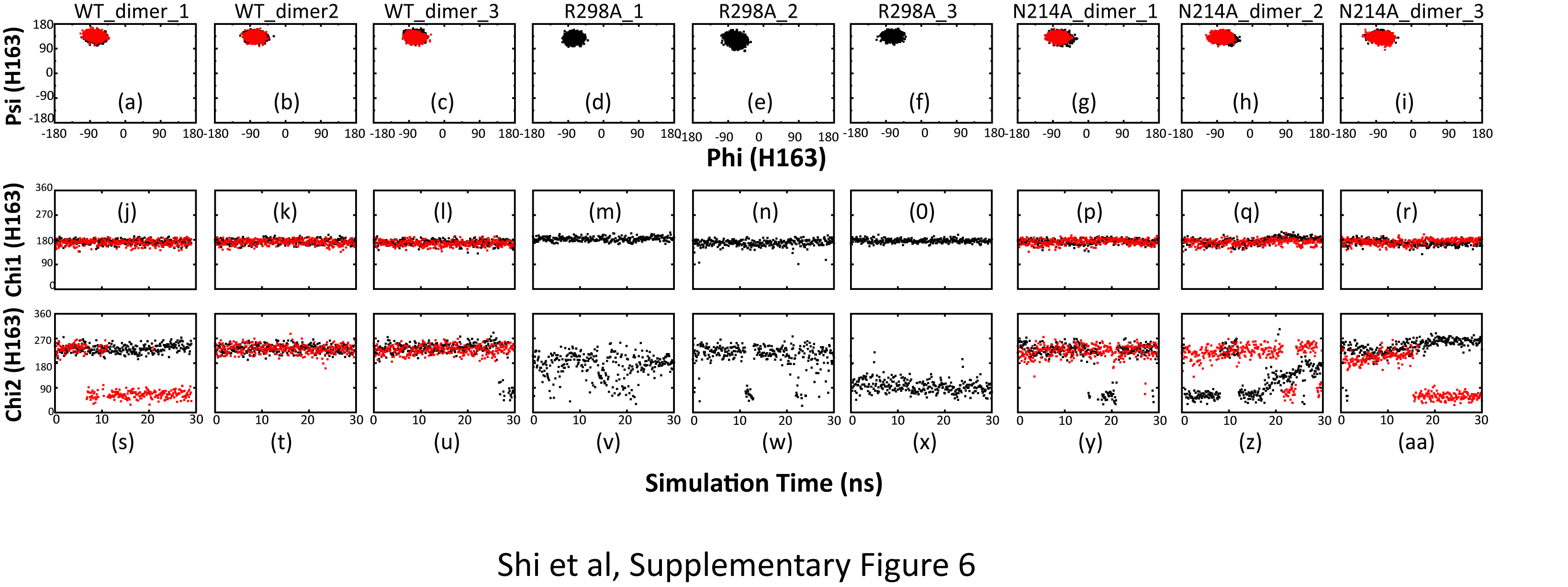

Supplement: Figure S6 — Dynamic behavior of His163. Ramachandran plots of His163 of WT (a–c); R298A (d–f) and N214A (g–i) in three independent simulations. Time-trajectories of the Chi1 dihedral angle of His163 of WT (j–l); R298A (m–o) and N214A (p–r). Trajectories of the Chi2 dihedral angle of His163 of WT (s–u); R298A (v–x) and N214A (y–aa). (0.34 MB TIF) [file pcbi.1001084.s006.tif]

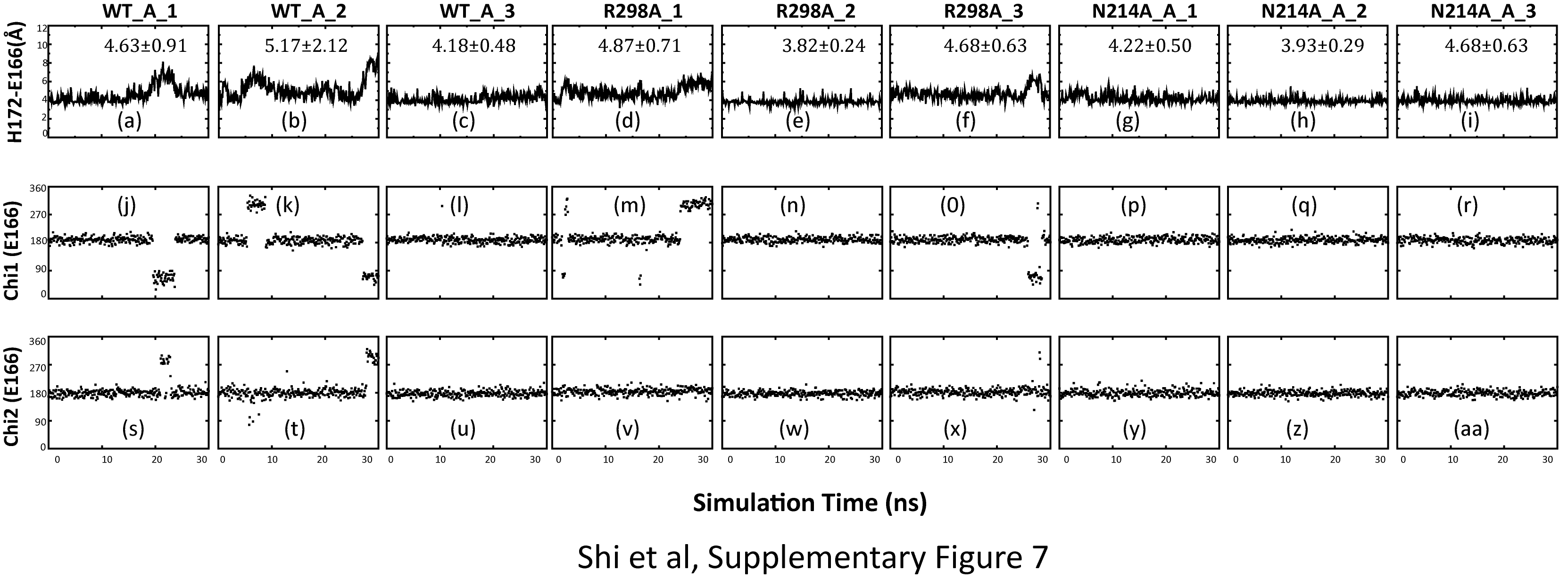

Supplement: Figure S7 — Dynamic behavior of the His172-Glu166 interaction in three monomers. Time-trajectories of the distances between the aromatic rings of His172 and Glu166 of the artificial WT (a–c); R298A (d–f) and artificial N214A (g–i) monomers in three independent simulations. Time-trajectories of the Chi1 dihedral angle of Glu166 of the artificial WT (j–l); R298A (m–o) and artificial N214A (p–r) monomers. Time-trajectories of the Chi2 dihedral angle of Glu166 of the artificial WT (s–u); R298A (v–x) and artificial N214A (y–aa) monomers. (4.11 MB TIF) [file pcbi.1001084.s007.tif]

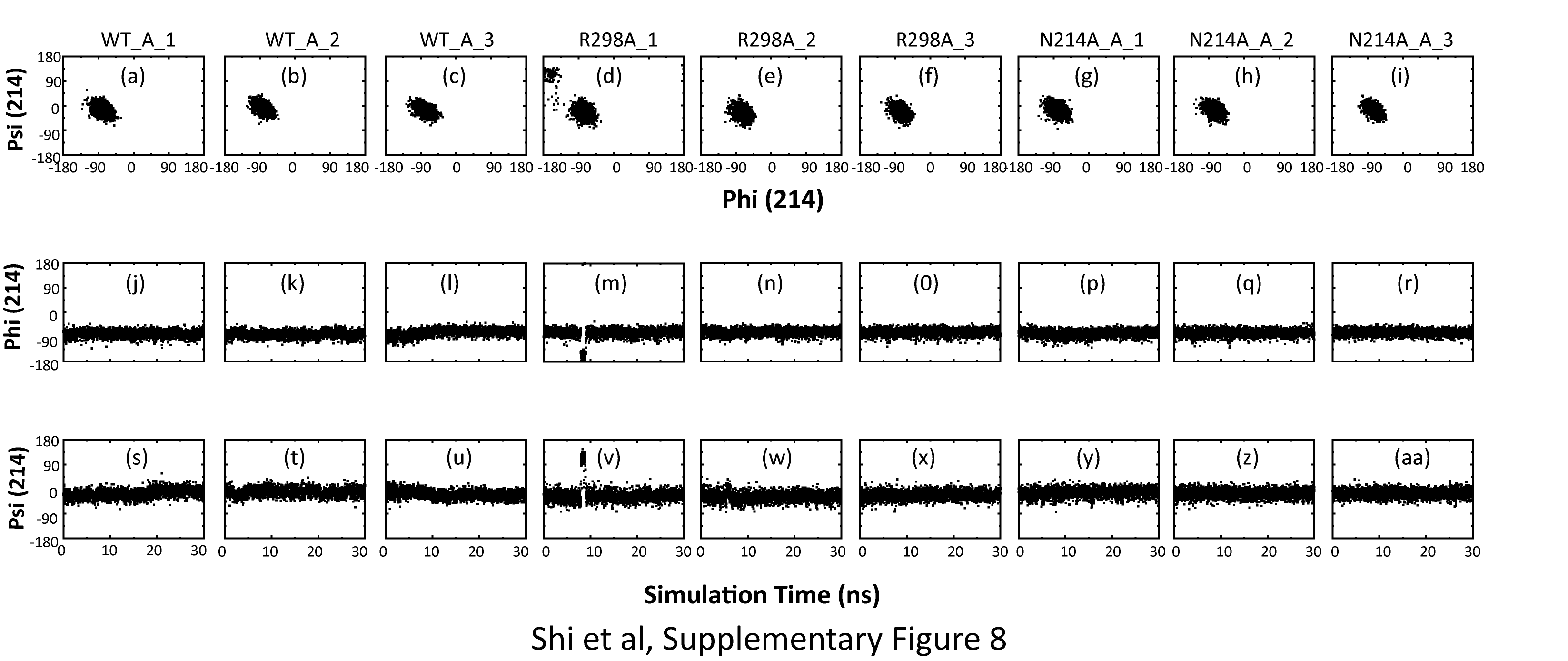

Supplement: Figure S8 — Dynamic behavior of the mutation site in three monomers. Ramachandran plots of the residues Asn214 for the artificial WT (a–c); R298A (d–f) and Ala214 for the artificial N214A (g–i) monomers in three independent simulations. Time-trajectories of the Phi dihedral angle of Asn214 of the artificial WT (j–l); R298A (m–o), and Ala214 for the artificial N214A (p–r) monomers. Time-trajectories of the Psi dihedral angle of Asn214 of the artificial WT (s–u); R298A (v–x), and Ala214 for the artificial N214A (y–aa) monomers. (4.65 MB TIF) [file pcbi.1001084.s008.tif]

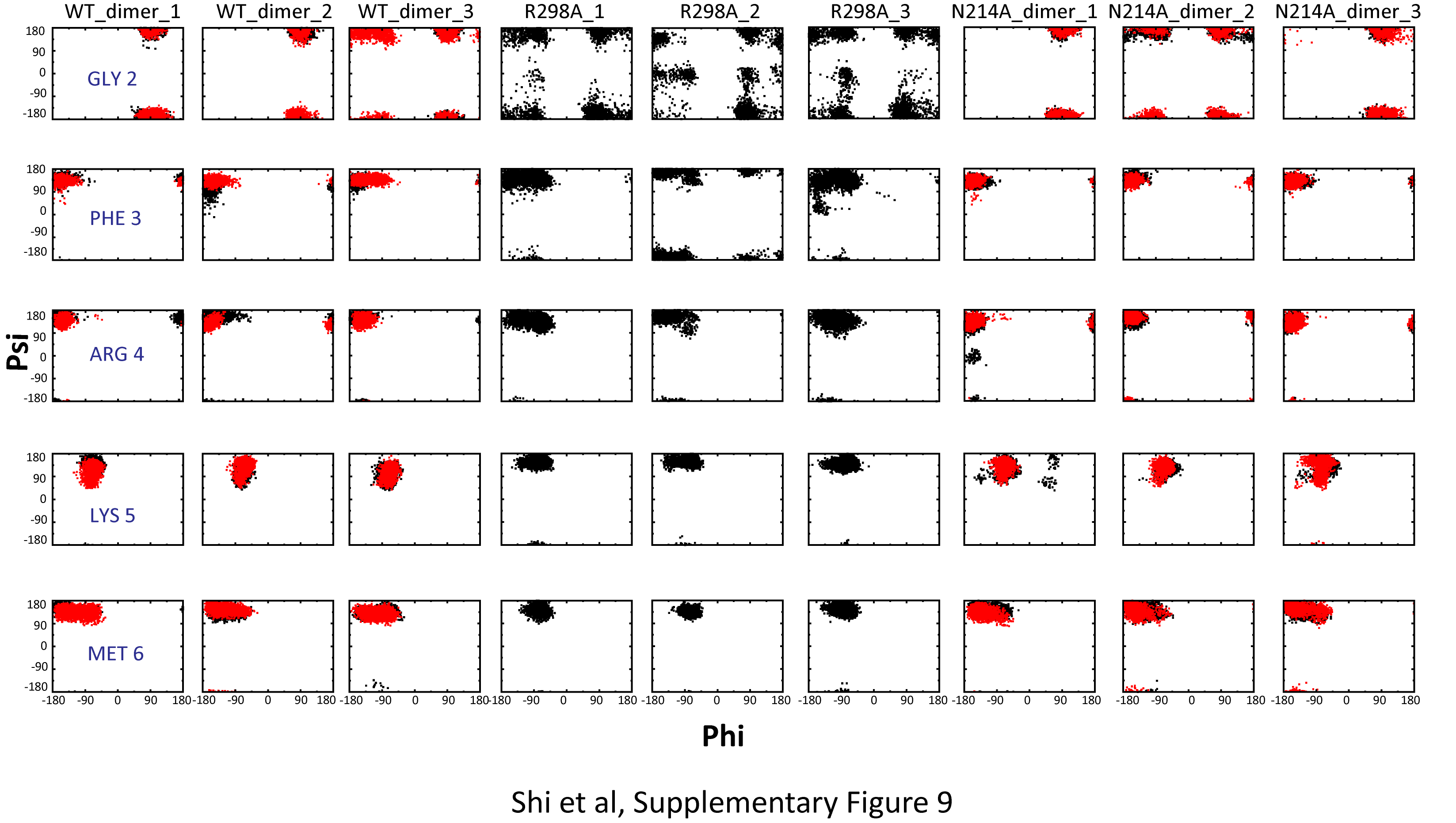

Supplement: Figure S9 — Ramachandran plots of the N-finger residues Gly2-Met6 in the three independent simulations for WT, R298A and N214A. (0.28 MB TIF) [file pcbi.1001084.s009.tif]
