# Supplementary material for: Dynamically-Driven Inactivation of the Catalytic Machinery of the SARS 3C-Like Protease by the N214A Mutation on the Extra Domain
Source: PLoS Comput Biol. 2011 Feb 24;7(2):e1001084. doi: 10.1371/journal.pcbi.1001084 (PMC3044768; doi:10.1371/journal.pcbi.1001084)
Supplement: Table S1 — Data collection and refinement statistics of the N214A crystal structure. (0.05 MB PDF) [file pcbi.1001084.s010.pdf]

Supplementary Table 1: Data collection and refinement statistics of crystal structures of SARS-CoV 3CLpro

	SARS-CoV 3CLpro mutant (N214A)
**PDB ID**	3M3S
**Data collection**	
Space group	$P2_1$
No. of molecule in ASU	2
Wavelength (Å)	1.5418
Resolution (Å)	66.48- 2.3 (2.4-2.3)[b]
Cell dimensions	
a (Å)	52.50
b (Å)	96.49
c (Å)	68.08
σ (°)	90
β (°)	102.47
γ (°)	90
Observed reflections	116159
Unique reflections	29514 (3511) [b]
R_{sym} or R_{merge}	0.111 (0.427) [b]
$I / \sigma I$	9.78 (1.95) [b]
Completeness (%)	99.9 (99.7) [b]
Redundancy	5.73 (3.22) [b]
**Refinement**	
Resolution (Å)	66.48-2.30(2.36-2.30) [b]
No. reflections	27953 (1798) [b]
R_{work} [c]	0.1959 (0.2609) [b]
No. reflections	26059 (1675) [b]
R_{free} [d]	0.2570 (0.3240) [b]
R.m.s. deviations[e]	
Bond lengths (Å)	0.005
Bond angles (°)	0.984
**Ramachandran plot**	
Favored, %	87.3
Allowed, %	11.2
Generously allowed, %	1.1
Disallowed, %	0.4

[a]One crystal was used for data collection.
[b]Values in parentheses are for the highest-resolution shell.
[c]$R_{work} = \Sigma(F_{obs} - F_{calc})/\Sigma F_{obs}$, where F_{calc} and F_{obs} are the calculated and observed structure factor amplitudes, respectively.
[d]R_{free} was calculated in the same manner as R_{work}, but for more than 1000 reflections chosen at random and omitted from refinement.
[e]RMSD, root mean square deviation.
