# Supplementary material for: Dynamically-Driven Inactivation of the Catalytic Machinery of the SARS 3C-Like Protease by the N214A Mutation on the Extra Domain
Source: PLoS Comput Biol. 2011 Feb 24;7(2):e1001084. doi: 10.1371/journal.pcbi.1001084 (PMC3044768; doi:10.1371/journal.pcbi.1001084)
Supplement: Table S2 — Average Occupancy (%) of hydrogen bonds associated with Asn214 and N-finger in WT/N214A. (0.06 MB PDF) [file pcbi.1001084.s011.pdf]

Supplementary Table 2. Average Occupancy (%) of Hydrogen Bonds Associated with Asn214 and N-finger in WT/N214A

Hydrogen bonds (long-range) associated with Asn214 in WT

Resid. No.	Resid ID	Atom	Resid. No.	Resid ID	Atom	Occupancy
214B	ASN	ND2	1B	SER	OG	7.118377
1B	SER	OG	214B	ASN	OD1	3.4492155
1B	SER	N	214B	ASN	OD1	2.071626333
214	ASN	ND2	1	SER	OG	1.797973
1B	SER	OG	214B	ASN	ND2	0.355228433
1	SER	N	214	ASN	OD1	0.1340215
1	SER	OG	214	ASN	OD1	0.0306986
2B	GLY	N	214B	ASN	OD1	11.85174567
2	GLY	N	214	ASN	OD1	10.01003667
214	ASN	ND2	2	GLY	O	2.107596667
2	GLY	N	214	ASN	ND2	0.186998667
214B	ASN	ND2	2B	GLY	O	0.131653167
214	ASN	ND2	2	GLY	N	0.125952033
214	ASN	ND2	300	CYS	SG	2.506147
214B	ASN	ND2	300B	CYS	SG	0.604939

Hydrogen bonds (long-range) associated with Asn214 in N214A

Resid. No.	Resid ID	Atom	Resid. No.	Resid ID	Atom	Occupancy
2	GLY	N	214	ALA	O	2.8497155

Inter-protomer hydrogen bonds with N-finger residues in WT

Resid. No.	Resid ID	Atom	Resid. No.	Resid ID	Atom	Occupancy
172B	HIE	NE2	1	SER	OG	24.56962

4B	ARG	NH1	137	LYS	O	1.2233874
172B	HIE	NE2	1	SER	O	0.286549333
139	SER	OG	6B	MET	SD	0.1890161
1	SER	OG	168B	PRO	O	0.180859
1B	SER	OG	140	PHE	O	0.0268394
1	SER	OG	170B	GLY	O	0.0267517
139B	SER	OG	4	ARG	N	0.0101744

Inter-protomer hydrogen bonds with N-finger residues in N214A

Resid. No.	Resid ID	Atom	Resid. No.	Resid ID	Atom	Occupancy
4	ARG	NH1	127B	GLN	O	85.4081
140	PHE	N	1B	SER	O	67.50826667
172	HIE	NE2	1B	SER	OG	29.902
139B	SER	OG	4	ARG	O	3.606305
137	LYS	NZ	3B	PHE	O	2.078125667
1B	SER	OG	167	LEU	O	1.488184167
139B	SER	OG	6	MET	SD	0.643268167
139	SER	OG	4B	ARG	O	0.516613667
1	SER	OG	167B	LEU	O	0.243308833
4	ARG	NH1	137B	LYS	O	0.151651667
170	GLY	N	1B	SER	OG	0.121654667
123B	SER	OG	6	MET	SD	0.009999

Inter-protomer hydrogen bonds with N-finger residues in both WT and N214A

Resid. No.	Resid ID	Atom	Resid. No.	Resid ID	Atom	Occupancy Diff. (WT-N214A)	WT Occupancy	N214A Occupancy
4	ARG	NE	137B	LYS	O	46.20704933	52.7880667	6.581017333
4	ARG	NH2	137B	LYS	O	27.90713817	29.8602767	1.9531385
4B	ARG	NE	137	LYS	O	25.48113333	68.3768667	42.89573333
4B	ARG	NH1	290	GLU	OE1	18.55989333	60.3407267	41.78083333
140B	PHE	N	1	SER	O	9.679566667	80.1941667	70.5146
1B	SER	N	166	GLU	OE2	9.289476667	16.0554667	6.76599
4B	ARG	NH1	127	GLN	O	8.689463333	13.0540267	4.364563333

1	SER	N	166B	GLU	OE2	6.593522667	12.6429233	6.049400667
1B	SER	N	166	GLU	OE1	6.282247	9.17195667	2.889709667
1	SER	OG	166B	GLU	OE1	4.632433333	40.0622333	35.4298
4	ARG	NH2	290B	GLU	OE2	2.737033333	47.3292333	44.5922
128	CYS	SG	4	ARG	NH1	1.2035615	1.53019567	0.326634167
3B	PHE	N	138	GLY	O	0.759419333	8.92528267	8.165863333
4B	ARG	NH2	137	LYS	O	0.64946	34.2011	33.55164
139	SER	OG	2B	GLY	O	0.462058	0.532051	0.069993
3	PHE	N	138B	GLY	O	0.0468325	1.50501883	1.458186333
4B	ARG	N	139	SER	OG	0.025438167	2.5351855	2.509747333
139B	SER	OG	2	GLY	O	-0.391277	0.158668	0.549945
1	SER	N	166B	GLU	OE1	-0.438028667	6.90290667	7.340935333
1B	SER	OG	166	GLU	OE1	-1.274194667	18.625492	19.89968667
4	ARG	NH2	290B	GLU	OE1	-1.496966667	50.0762	51.57316667
172	HIE	NE2	1B	SER	O	-1.678519167	0.36127883	2.039798
4B	ARG	NH1	290	GLU	OE2	-2.815166667	33.2878667	36.10303333
4B	ARG	NH2	290	GLU	OE2	-4.807703333	53.1431967	57.9509
1	SER	N	140B	PHE	O	-4.871016667	12.6205633	17.49158
4	ARG	NH1	290B	GLU	OE2	-5.829366667	48.7035	54.53286667
4	ARG	N	139B	SER	OG	-10.81179967	0.210417	11.02221667
1B	SER	N	140	PHE	O	-12.722006	5.34952733	18.07153333
1	SER	OG	166B	GLU	OE2	-13.3032	20.9050367	34.20823667
4	ARG	NH1	290B	GLU	OE1	-14.84663333	35.41	50.25663333
4B	ARG	NH2	290	GLU	OE1	-15.74603333	29.7494333	45.49546667
1B	SER	OG	166	GLU	OE2	-19.77519683	3.94906983	23.72426667
